# Isolation and characterization of *Chlorella* sp. mutants with enhanced thermo- and CO_2_ tolerances for CO_2_ sequestration and utilization of flue gases

**DOI:** 10.1186/s13068-019-1590-9

**Published:** 2019-10-19

**Authors:** Hsiang-Hui Chou, Hsiang-Yen Su, Xiang-Di Song, Te-Jin Chow, Chun-Yen Chen, Jo-Shu Chang, Tse-Min Lee

**Affiliations:** 1 Department of Biotechnology, Fooyin University, Kaohsiung, 83102 Taiwan; 2 Department of Biological Sciences, National Sun Yat-sen University, Kaohsiung, 80424 Taiwan; 3 China-Latin America Joint Laboratory for Clean Energy and Climate Change, School of Chemical Engineering and Energy Technology, Dongguan University of Technology, Dongguan, 523808 China; 4 University Center of Bioscience and Biotechnology, National Cheng Kung University, Tainan, 70146 Taiwan; 5 Department of Chemical Engineering, National Cheng-Kung University, Tainan, 70146 Taiwan; 6 Research Center for Energy Technology and Strategy, National Cheng Kung University, Tainan, 70146 Taiwan; 7 Department of Marine Biotechnology and Resources, National Sun Yat-sen University, Kaohsiung, 80424 Taiwan

**Keywords:** Flue gas, Microalgae, *Chlorella* sp., Thermo-tolerance

## Abstract

**Background:**

The increasing emission of flue gas from industrial plants contributes to environmental pollution, global warming, and climate change. Microalgae have been considered excellent biological materials for flue gas removal, particularly CO_2_ mitigation. However, tolerance to high temperatures is also critical for outdoor microalgal mass cultivation. Therefore, flue gas- and thermo-tolerant mutants of *Chlorella vulgaris* ESP-31 were generated and characterized for their ability to grow under various conditions.

**Results:**

In this study, we obtained two CO_2_- and thermo-tolerant mutants of *Chlorella vulgaris* ESP-31, namely, 283 and 359, with enhanced CO_2_ tolerance and thermo-tolerance by using *N*-methyl-*N*-nitro-*N*-nitrosoguanidine (NTG) mutagenesis followed by screening at high temperature and under high CO_2_ conditions with the w-zipper pouch selection method. The two mutants exhibited higher photosynthetic activity and biomass productivity than that of the ESP-31 wild type. More importantly, the mutants were able to grow at high temperature (40 °C) and a high concentration of simulated flue gas (25% CO_2_, 80–90 ppm SO_2_, 90–100 ppm NO) and showed higher carbohydrate and lipid contents than did the ESP-31 wild type.

**Conclusions:**

The two thermo- and flue gas-tolerant mutants of *Chlorella vulgaris* ESP-31 were useful for CO_2_ mitigation from flue gas under heated conditions and for the production of carbohydrates and biodiesel directly using CO_2_ from flue gas.

## Background

The threat of global warming caused by the increasing levels of CO_2_ in the atmosphere due to the combustion of fossil fuels is of great concern [1]. Flue gases emitted from industrial plants containing high concentrations of CO_2_, NO_X_ and SO_X_ are mostly responsible for global CO_2_ emissions [2, 3]. The use of microalgae for biological CO_2_ mitigation and flue gas cleaning (removal) has attracted global attention because (1) microalgae do not compete directly with food crops for land or water; (2) they can fix CO_2_ via photosynthesis at much higher rates than terrestrial higher plants can [4–7]; and (3) the microalgal biomass obtained by the consumption of CO_2_ is a very useful feedstock for the production of biofuels and many valuable chemicals [8, 9].

In general, typical flue gas emitted from combustion sources contains 10–15% CO_2_, and coupling microalgae cultivation with CO_2_ generated by industrial plants has the potential to reduce the cost of flue gas pretreatment of industrial plants and industrial-scale microalgae production [10, 11]. However, the presence of high concentrations of CO_2_, NO_X_, SO_X_, and other impurities of industrial flue gases inhibits the growth of most microalgae [12, 13]. Therefore, the generation and selection of fast-growing microalgal strains or mutants with high CO_2_ fixation efficiency and the ability to tolerate high concentrations of CO_2_, NO_X_, and SO_X_ in flue gases will improve the efficiency and cost-effectiveness of microalgal flue gas CO_2_ mitigation processes [12, 14, 15].

The tolerance to high temperatures is also critical for outdoor microalgal mass cultivation. The optimal growth temperature for most algae is approximately 20–30 °C [12, 16], and the high temperatures during outdoor cultivation suppress the growth of most microalgae.

In subtropical regions such as Taiwan, the temperature of microalgal culture medium in photobioreactors (PBRs) frequently surpasses 40 °C due to sunlight irradiation during the daytime [17]. Thus, using microalgae strains that can tolerate high temperatures would significantly reduce the cooling costs for outdoor cultivation. Although many thermo-tolerant microalgal strains have been isolated from hot springs [18–20], they may not be able to tolerate the high levels of CO_2_, SO_X_, and NO_X_ in flue gases.

A lipid-rich green microalga, *Chlorella vulgaris* ESP-31, exhibits 30–31 mg/L/day lipid productivity and 35–44% lipid content, respectively, when grown under phototrophic and photoheterotrophic conditions using CO_2_ and acetic acid as the carbon source, respectively [21]. However, when grown outdoors, the biomass productivity obtained from phototrophic and photoheterotrophic conditions was only 0.07 and 0.11 g/L/day, respectively, compared to 0.26 and 0.20 g/L/day obtained under indoor cultivation conditions [21].

In this study, *C. vulgaris* ESP-31 was subjected to *N*-methyl-*N*′-nitro-*N*-nitrosoguanidine (NTG) mutagenesis [22] and then screened for mutants tolerant to high temperature and high CO_2_ concentration. Two mutants of *C. vulgaris* ESP-31, mutants 283 and 359, with the ability to tolerate heat and high levels of CO_2_, NO, and SO_2_ were isolated and characterized. These mutants showed the ability to grow at high temperature (40 °C) in simulated flue gas containing 25% CO_2_/air, 80–90 ppm SO_2_, and 90–100 ppm NO. The mutants also showed higher carbohydrate and lipid contents than those of the *C. vulgaris* ESP-31 wild type, and they may be useful for CO_2_ mitigation and the production of carbohydrates and biodiesel by directly using CO_2_ from flue gas.

## Results

### Isolation of *Chlorella vulgaris* ESP-31 mutants under the conditions of high temperature and high CO_2_ concentration

Over 500 potential mutants of *C. vulgaris* ESP-31 were obtained after NTG mutagenesis, all of which were screened for the tolerance of high temperature (40 °C) and high CO_2_ concentration (15% CO_2_/air) using the w-zipper pouch selection method. Of them, two mutants (namely, mutant 283 and mutant 359) showed better growth than the wild type under 40 °C and 15% CO_2_/air. The two mutants and wild type were grown at 28 °C in BG-11 medium under two different CO_2_ concentrations (5 and 25% CO_2_/air) and 150 μmol m^−2^ s^−1^ for 4 days, followed by detection and quantification of the biomass, maximum biomass productivity and net photosynthetic rates. The results showed that the biomass and net photosynthetic O_2_ evolution rates of mutants 283 and 359 were increased when compared with those of the wild type.

**Fig. 1:**
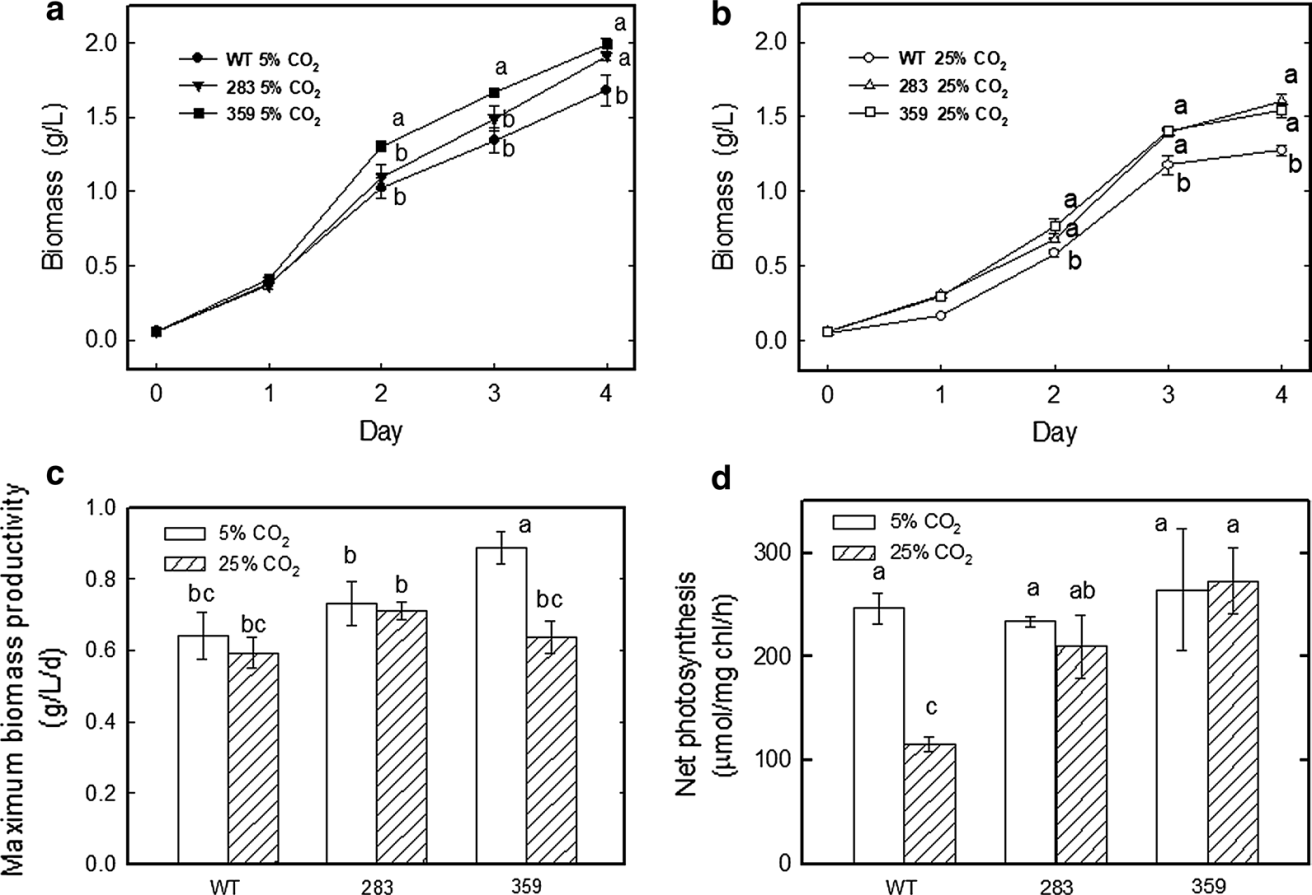
Effects of different CO_2_ concentrations on the growth and photosynthesis rates of the wild type and mutants 283 and 359 of *Chlorella vulgaris* ESP-31. **a** Growth curves under 5% CO_2_. **b** Growth curves under 25% CO_2_. **c** Biomass productivity. **d** Net photosynthesis rates. A 50-mL culture of each strain aerated with different CO_2_ (0.1 vvm) was grown in BG-11 at 28 °C under 12 h light–dark (light intensity 150 μmol m^−2^ s^−1^) conditions for 4 days. Data shown are the means (along with the standard deviations) of at least two independent experiments

When aerated with 5% CO_2_/air, compared to the wild type, mutants 283 and 359 exhibited biomass increases of 14% and 18%. The biomass values were measured as 1.68, 1.91, and 1.99 g/L for the wild type and mutants 283 and 359, respectively (Fig. 1a). As a consequence, the maximum biomass productivity of strains 283 and 359 was increased by 14% and 39%, respectively, when compared to that of the wild type, with a maximum biomass productivity of 0.64, 0.73 and 0.89 g/L/day for the wild type and mutants 283 and 359, respectively, after 4 days (Fig. 1c).

When grown under 25% CO_2_/air, compared with the wild type, mutants 283 and 359 exhibited biomass increases of 25% and 20%, respectively. The biomass was measured as 1.28, 1.61, and 1.54 g/L for the wild type and mutants 283 and 359, respectively (Fig. 1b). Consistently, the maximum biomass productivity of mutants 283 and 359 was increased by 20% and 8%, respectively, when compared to that of the wild type, with a maximum biomass productivity of 0.59, 0.71 and 0.64 g/L/day for the wild-type, 283 and 359 strains, respectively, after 4 days (Fig. 1c).

The net photosynthetic O_2_ evolution rate was determined in mutants 283 and 359 in response to different CO_2_ concentrations (5 and 25% CO_2_/air). As shown in Fig. 1d, the net photosynthetic O_2_ evolution rates of mutants 283 and 359 were similar to those of the wild type with 5% CO_2_/air. However, when grown in medium aerated with 25% CO_2_/air, the wild type presented inhibited net photosynthetic O_2_ evolution rates compared with those under 5% CO_2_/air. In contrast, the values for mutants 283 and 359 remained almost the same as those with 5% CO_2_/air aeration. Therefore, under 25% CO_2_/air, the net photosynthetic O_2_ evolution rates were increased by 1.8- to 2.4-fold in the mutants in comparison with the wild type. The net photosynthetic O_2_ evolution rates were measured as 115.7, 210.4, and 272.9 μmol/mg chl/h for the wild type and mutants 283 and 359, respectively (Fig. 1d).

Taken together, when grown at 28 °C in 5% CO_2_/air under 150 μmol m^−2^ s^−1^ light intensity, the growth and photosynthetic efficiency of mutants 283 and 359 were significantly higher than those of the wild type. As the CO_2_ level increased to 25%, both the growth and photosynthetic efficiency of these two mutants and the wild type were decreased. However, under 25% CO_2_ conditions, both the growth and photosynthetic efficiency of the two mutants were much higher than those of the wild type. This result indicated that mutants 283 and 359 can tolerate higher levels of CO_2_ than can the wild type.

### Effects of high temperature on the cell growth and photosynthesis rates of *Chlorella vulgaris* ESP-31 mutants

**Fig. 2:**
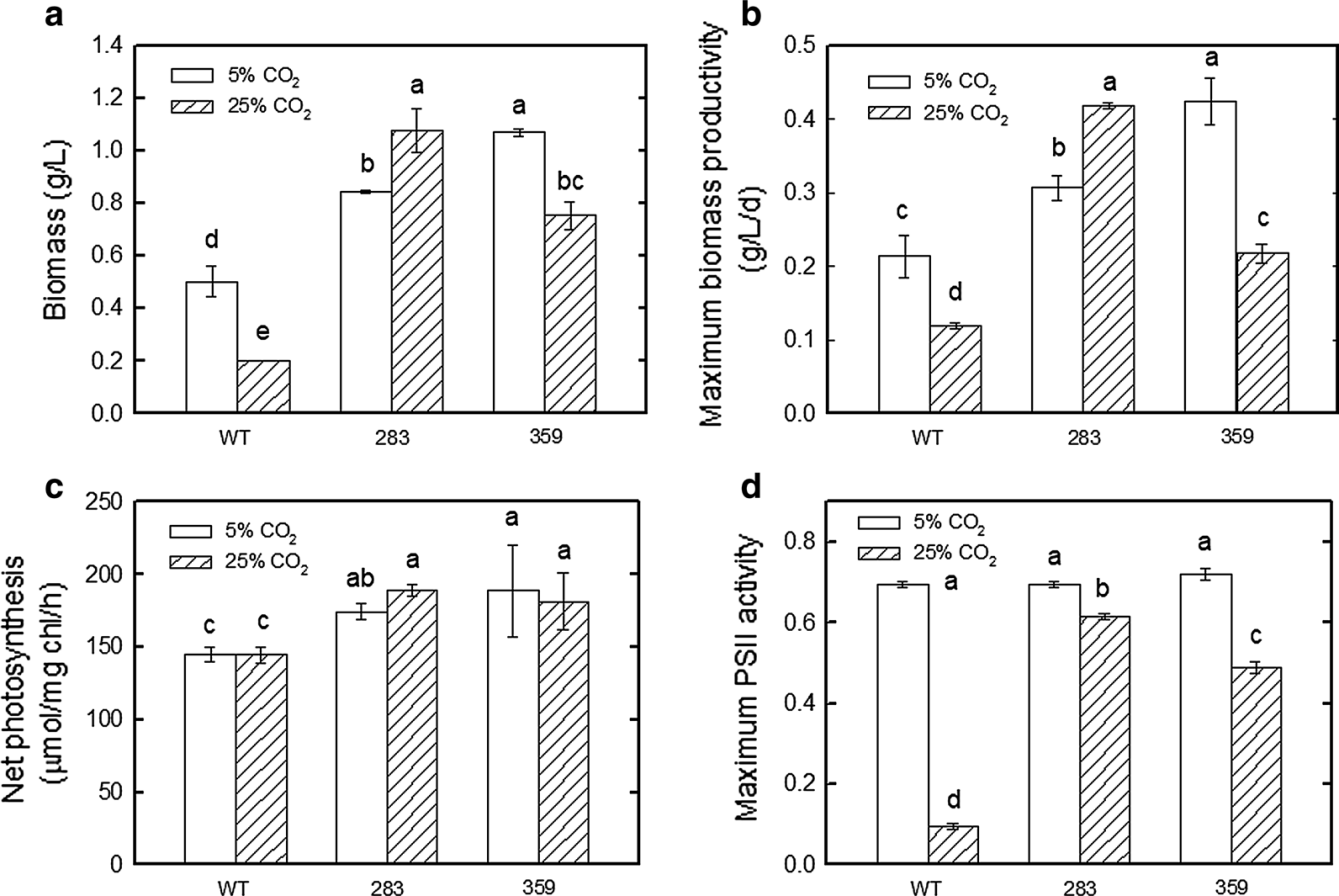
Effects of high temperature and different CO_2_ concentrations on the growth and photosynthetic rates of the wild type and mutants 283 and 359 of *Chlorella vulgaris* ESP-31. **a** The growth performance under different CO_2_ concentrations (5 and 25% CO_2_/air) at 40 °C. **b** Biomass productivity. **c** Net photosynthesis rates. (D) Maximum PSII activity. A 50-mL culture of each strain aerated with different CO_2_ (0.1 vvm) was grown in BG-11 at 40 °C under 12 h light–dark (light intensity 150 μmol m^−2^ s^−1^) conditions for 4 days. Data shown are the means (along with the standard deviations) of at least two independent experiments

High temperature impacts the growth and photosynthesis of algae in outdoor cultivation systems [23, 24]. The temperatures in the closed PBRs usually surpass 40–45 °C without temperature control in tropical and subtropical regions. Therefore, high temperatures have become an issue for outdoor cultivation. In the present study, to examine the combined effects of high CO_2_ concentration and high temperature on cell growth, the two mutants (mutants 283 and 359) and the wild type were grown at 40 °C in BG-11 medium under two different CO_2_ concentrations (5 and 25% CO_2_/air) and 150 μmol m^−2^ s^−1^ for 4 days, separately, followed by detection of the biomass, maximum biomass productivity, net photosynthetic rates and maximum PSII activity. The biomass and net photosynthetic O_2_ evolution rates of mutants 283 and 359 were increased in comparison with those of the wild type (Fig. 2).

When aerated with 5% CO_2_/air, compared with the wild type, mutants 283 and 359 exhibited biomass increases of 1.7- and 2.1-fold. The values of biomass were measured as 0.50, 0.84, and 1.07 g/L for the wild type and mutants 283 and 359, respectively (Fig. 2a). Meanwhile, the maximum biomass productivity of mutants 283 and 359 was increased by 1.5- and twofold, respectively, when compared to that of the wild type, with a biomass productivity of 0.21, 0.31 and 0.42 g/L/day for the wild type and mutants 283 and 359, respectively, after 4 days (Fig. 2b).

When grown at 40 °C in the medium aerated with 5% CO_2_/air, compared with the wild type, mutants 283 and 359 exhibited net photosynthetic O_2_ evolution rate increases of 20.3% and 30.2%, respectively. The photosynthetic O_2_ evolution rates were measured as 145.1, 174.5, and 188.9 μmol/mg chl/h for the wild type and mutants 283 and 359, respectively (Fig. 2c).

When aerated with 25% CO_2_/air, mutants 283 and 359 exhibited biomass increases of 5.4- and 3.8-fold, respectively. The biomass was measured as 0.20, 1.08 and 0.75 g/L for the wild type and mutants 283 and 359, respectively (Fig. 2a). The maximum biomass productivity of mutants 283 and 359 increased by 3.5-fold and 1.8-fold, respectively, when compared to that of the wild type, with a biomass productivity of 0.12, 0.42 and 0.22 g/L/day for the wild type and mutants 283 and 359, respectively, after 4 days (Fig. 2b). Although the mutant strain 359 showed a lower biomass under the 25% CO_2_ condition than under the 5% CO_2_ condition, strain 283 showed a higher biomass under the 25% CO_2_ condition than under the 5% CO_2_ condition. When grown at 40 °C in the medium aerated with 25% CO_2_/air, compared with the wild type, mutants 283 and 359 exhibited photosynthetic O_2_ evolution rate increases of 30% and 25.4%. The photosynthetic O_2_ evolution rates were measured as 144.8, 188.9, and 181.5 μmol/mg chl/h for the wild type and mutants 283 and 359, respectively (Fig. 2c).

The PSII activities of mutants 283 and 359 were determined in response to 40 °C under different CO_2_ concentrations (5 and 25% CO_2_/air). As shown in Fig. 2d, the growth of mutants 283 and 359 was similar to that of the wild type with 5% CO_2_/air. However, when mutants 283 and 359 were grown in the medium aerated with 25% CO_2_/air, the maximum PSII activity of remained stable. The maximum PSII activity was measured as 0.095, 0.615 and 0.49 for the wild type and mutants 283 and 359, respectively (Fig. 2d).

### Effects of simulated flue gas and high temperature on the cell growth, photosynthesis rates, and carbohydrate and lipid levels of *Chlorella vulgaris* ESP-31 mutants under indoor conditions

To investigate the effects of simulated flue gas (25% CO_2_, 80–90 ppm SO_2_, 90–100 ppm NO) and high temperature on cell growth, the wild type and mutants 283 and 359 were grown at 40 °C in ¼ N BG11 medium (0.375 g/L NaNO_3_) aerated with the simulated flue gas (0.1 vvm) under 150 μmol m^−2^ s^−1^ light intensity, followed by measurement of the biomass, biomass productivity, net photosynthetic rates, maximum PSII activity and nitrate concentration.

**Fig. 3:**
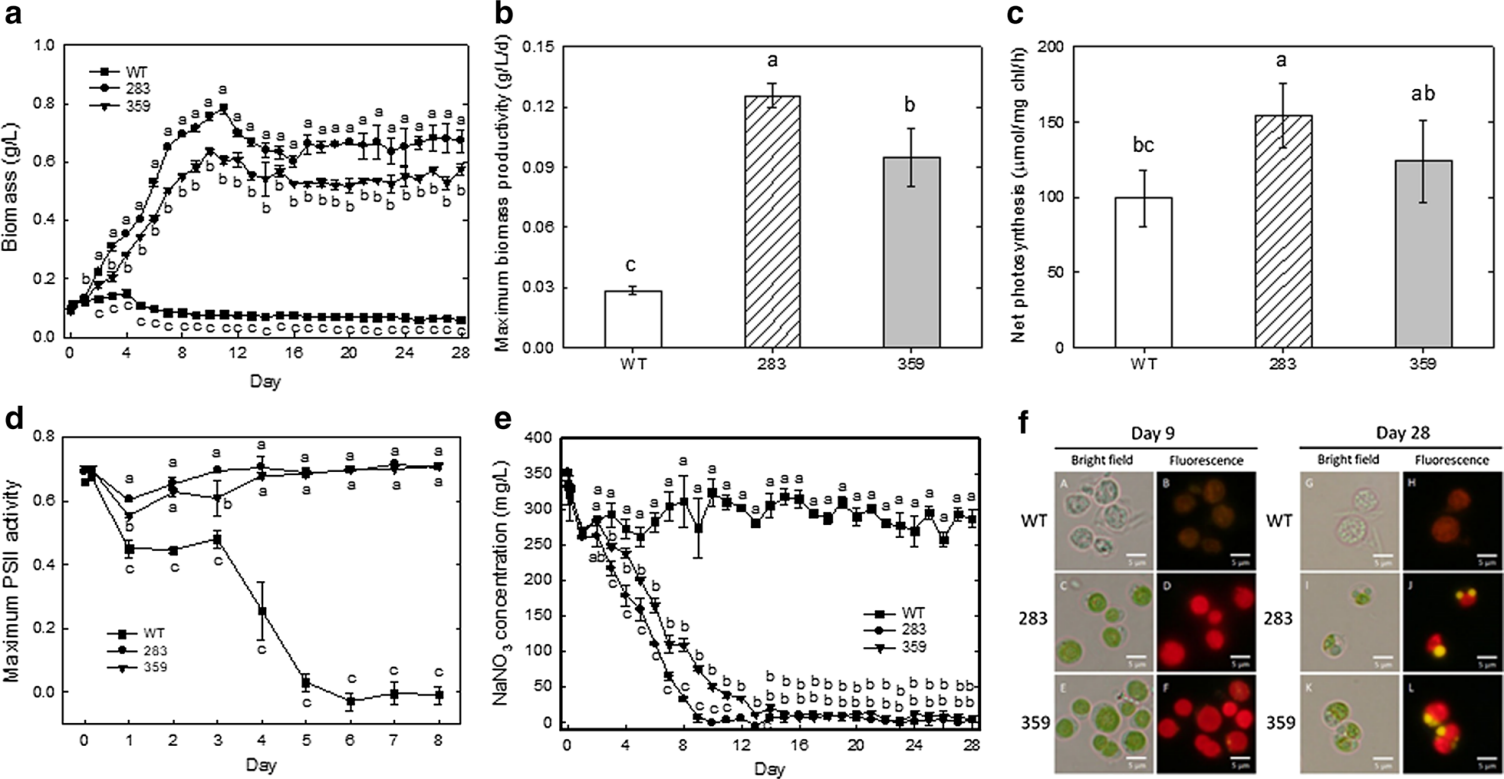
Effects of simulated flue gas and high temperature on the growth and photosynthetic rates of the wild type and mutants 283 and 359 of *Chlorella vulgaris* ESP-31. **a** Growth curves. **b** Biomass productivity. **c** Net photosynthesis rates. **d** Maximum PSII activity. **e** Nitrate concentration. **f** Nile red staining. Bright field: A, C, E, G, I, K. Fluorescence: B, D, F, H, J, L. An 800-mL culture of each strain aerated with simulated flue gas (0.1 vvm) was grown in ¼ N BG-11 medium (containing 0.375 g/L NaNO_3_) at 40 °C with a speed at 150 rpm under a light cycle consisting of 12 h of light with 150 μmol m^−2^ s^−1^ and then aeration with air (0.1 vvm) at 28 °C with a speed at 150 rpm of 12 h of dark conditions for 9 days. Next, the cells were cultivated by aeration with air (0.1 vvm) at 28 °C with a speed at 150 rpm and a light/dark cycle of 12 h/12 h at a light intensity of 150 μmol m^−2^ s^−1^ until 28 days. Data shown are the means (along with the standard deviations) of at least two independent experiments

When aerated with simulated flue gas (25% CO_2_, 80–90 ppm SO_2_, 90–100 ppm NO) and grown at 40 °C, mutants 283 and 359 showed a better survival rate than the wild type; the values of biomass were measured as 0.08, 0.72 and 0.58 g/L for the wild type and mutants 283 and 359, respectively, after 9 days of aeration with simulated flue gas and grown at 40 °C (Fig. 3a). The maximum biomass productivity of mutants 283 and 359 increased by 4.5- and 3.4-fold, respectively, when compared to that of the wild type, with a biomass productivity of 0.028, 0.126 and 0.095 g/L/day for the wild type and mutants 283 and 359, respectively (Fig. 3b). The net photosynthetic O_2_ evolution rates of mutants 283 and 359 increased by 55.2% and 24.5% after 2 days of cultivation. The photosynthetic O_2_ evolution rates were measured as 99.4, 154.3 and 123.8 μmol/mg chl/h for the wild type and mutants 283 and 359, respectively (Fig. 3c). As shown in Fig. 3d, when mutants 283 and 359 were grown at 40 °C, the maximum PSII activity remained stable in comparison with that of the wild type (Fig. 3d).

After 9 days of cultivation, mutants 283 and 359 showed that the nitrogen source concentration in the medium was depleted (Fig. 3e). Figure 3f shows images of cells of the wild type and mutants 283 and 359 after 9 days in simulated flue gas. The use of fluorescent Nile red for cellular lipid measurement showed that Nile red fluorescence cannot be found during simulated flue gas injection (9 days) while algal cells were stained with Nile red fluorescence after air aeration for 28 days. The simulated flue gases containing NO might influence the lipid accumulation of cells. Therefore, to promote the lipid accumulation, cultures were switched to air aeration after 9 days of growth in simulated flue gas until 28 days.

After 28 days of culture, the carbohydrate content in mutants 283 and 359 was 29.98 and 24.02%, respectively, and the lipid content in the wild type and mutants 283 and 359 was 3.72, 17.84 and 17.49%, respectively (Table 1). The carbohydrate production of mutants 283 and 359 was 234.53 and 152.71 mg/L, respectively, and the lipid production of the wild type and mutants 283 and 359 was 5.57, 139.31 and 111.03 mg/L, respectively (Table 1).

**Table 1:** Carbohydrate and lipid production by *Chlorella vulgaris* ESP-31 mutants grown in simulated flue gas and high temperature

Strain	Carbohydrate content (%)^a^	Carbohydrate production (mg/L)^b^	Lipid content (%)^c^	Lipid production (mg/L)^d^
Wild type	–	–	3.72 ± 0.002	5.57 ± 0.70
283	29.98 ± 1.71	234.53 ± 18.39	17.84 ± 0.016**	139.31 ± 9.69**
359	24.02 ± 1.04	152.71 ± 4.23	17.49 ± 0.030*	111.03 ± 17.27*

Each datum indicates the mean ± SD from two experiments

* *P* < 0.05 and **P < 0.01 were statistically significant as compared with *Chlorella vulgaris* ESP-31 wild type

–, no data because the cells were dead

^a^Carbohydrate content (%) = (carbohydrate dry weight/biomass dry weight) × 100%

^b^Carbohydrate production (mg/L) = (Max. biomass production × carbohydrate content)/100 × 1000

^c^Lipid content (%) = (lipid dry weight/biomass dry weight) × 100%

^d^Lipid production (mg/L) = (Max. biomass production × lipid content)/100 × 1000

### Cell growth, photosynthesis, and carbohydrate and lipid production performance of *Chlorella vulgaris* ESP-31 mutants grown in simulated flue gas under outdoor culture conditions

The growth performance of *C. vulgaris* ESP-31 mutant 283 and 359 strains in the field was examined in a 1800-mL outdoor PBR from September 6-October 6, 2017, in the School of Environment and Life Sciences, Fooyin University, Kaohsiung, Taiwan (22°60′15.65″N, 120°38′89.70″E). The wild-type, 283 and 359 strains were grown in 1800 mL ¼ BG-11 medium under the conditions of intermittent simulated flue gas aeration (25% CO_2_, 90–100 ppm NO, 80–90 ppm SO_2_) (0.1 vvm) at daytime and then aeration with air (0.1 vvm) at night for 6 days. Next, cells were cultivated by aeration with air (0.1 vvm) until 30 days, followed by comparison of the biomass, nitrate concentration and net photosynthetic rates.

**Fig. 4:**
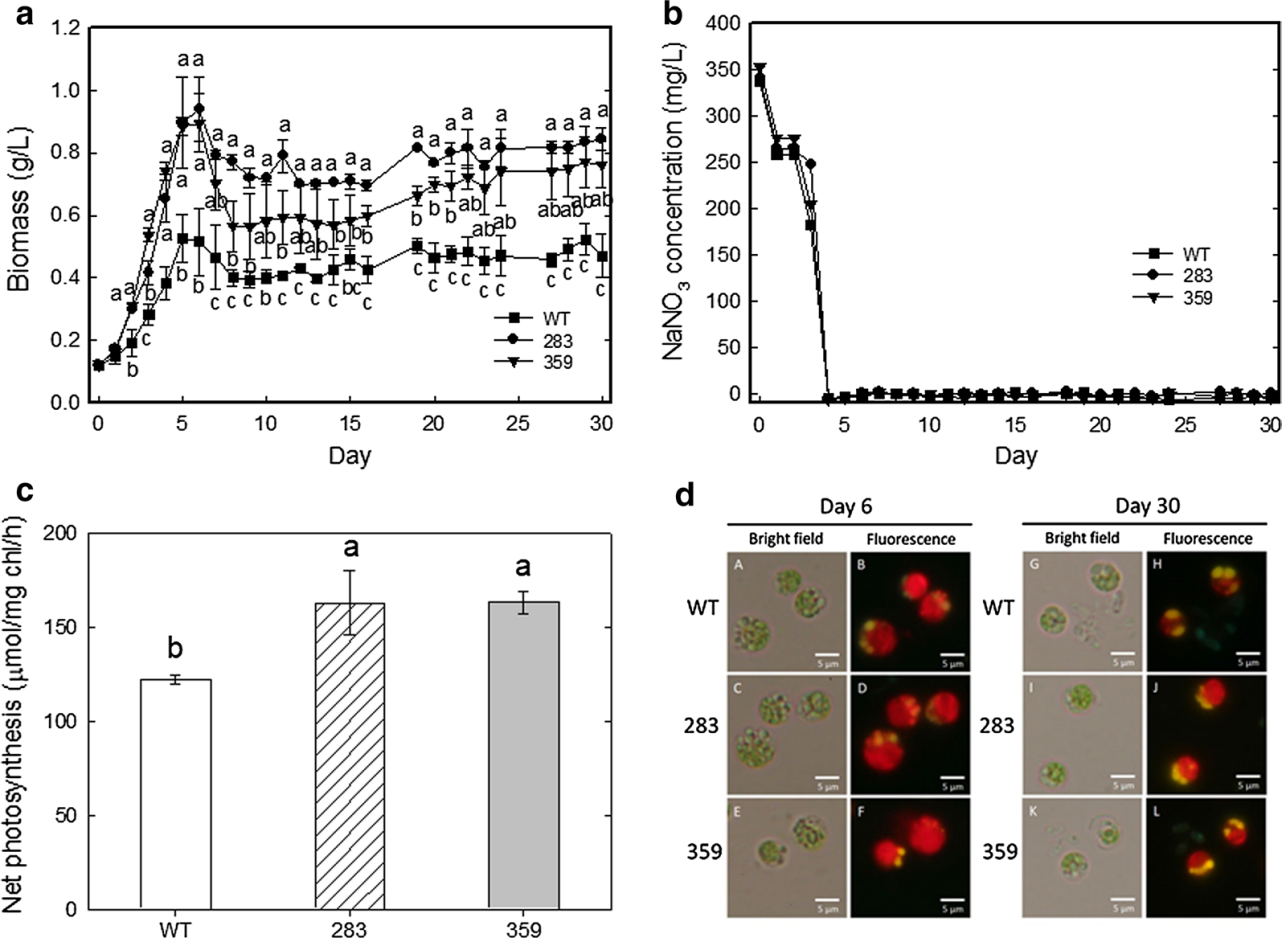
Growth and photosynthesis rates of the wild type and mutants 283 and 359 of *Chlorella vulgaris* ESP-31 in a closed photobioreactor aerated with simulated flue gas under outdoor cultivation. **a** Growth curves, **b** Nitrate concentration, **c** net photosynthesis rate, **d** Nile red staining. Bright field: A, C, E, G, I, K. Fluorescence: B, D, F, H, J, L. A 1800-mL culture of each strain aerated with simulated flue gas (0.1 vvm) was grown in ¼ N BG-11 medium (containing 0.375 g/L NaNO_3_) at daytime and then aerated with air (0.1 vvm) at night for 6 days. Next, cells were cultivated by aeration with air (0.1 vvm) until 30 days. Data shown are the means (along with the standard deviations) of at least two independent experiments

When grown in simulated flue gas under outdoor culture conditions, compared with the wild type, strains 283 and 359 exhibited maximum biomass increases of 1.8-fold and 1.7-fold, respectively. The maximum biomass was measured as 0.53, 0.94 and 0.89 g/L for the wild-type, 283 and 359 strains, respectively (Fig. 4a). As a consequence, the nitrate concentration in the medium was depleted after 4 days (Fig. 4b).

The net photosynthetic O_2_ evolution rates of strains 283 and 359 were increased by 1.3- and 1.3-fold in comparison with that of the wild type after 2 days of culture. The photosynthetic O_2_ evolution rates were measured as 122.5, 163.0 and 163.3 μmol/mg chl/h for the wild-type, 283 and 359 strains, respectively (Fig. 4c).

The carbohydrate and lipid contents of *C. vulgaris* ESP-31 wild type, 283 and 359 strain cultures that were aerated with simulated flue gas (25% CO_2_, 90–100 ppm NO, 80–90 ppm SO_2_) are shown in Table 2. After 30 days of cultivation, the carbohydrate and lipid contents were similar among the wild-type, 283 and 359 strains. The carbohydrate content in the wild type and mutants 283 and 359 was 28.98%, 27.28% and 29.78%, respectively, and the lipid content was 14.08%, 12.24% and 13.78% for the wild-type, 283 and 359 strains, respectively. Figure 4e also shows that the algal cells of the wild-type, 283 and 359 strains were stained by Nile red fluorescence at 6 day before the change to air aeration, and also at 30 day. The carbohydrate production of the wild-type, 283 and 359 strains was 153.36, 255.83 and 266.60 mg/L, respectively, and the lipid production of the wild-type, 283 and 359 strains was 75.10, 115.81 and 123.61 mg/L, respectively (Table 2).

**Table 2:** Carbohydrate and lipid production by *Chlorella vulgaris* ESP-31 mutants grown in closed outdoor PBR aerated with simulated flue gas

Strain	Carbohydrate content (%)^a^	Carbohydrate production (mg/L)^b^	Lipid content (%)^c^	Lipid production (mg/L)^d^
Wild type	28.98 ± 1.60	153.36 ± 30.65	14.08 ± 2.32	75.1 ± 23.01
283	27.28 ± 0.70	255.83 ± 21.78	12.24 ± 1.76	115.81 ± 29.27
359	29.78 ± 0.25	266.60 ± 30.02	13.78 ± 0.73	123.61 ± 19.36

Each datum indicates the mean ± SD from two experiments

^a^Carbohydrate content (%) = (carbohydrate dry weight/biomass dry weight) × 100%

^b^Carbohydrate production (mg/L) = (Max. biomass production × carbohydrate content)/100 × 1000

^c^Lipid content (%) = (lipid dry weight/biomass dry weight) × 100%

^d^Lipid production (mg/L) = (Max. biomass production × lipid content)/100 × 1000

### Comparison between indoor and outdoor conditions

The results in Table 3 show the maximum biomass, maximum biomass productivity, CO_2_ biofixation rate and specific growth rate of *C. vulgaris* ESP-31 mutants grown under indoor and outdoor conditions with simulated flue gas. In indoor cultivations, the maximum biomass of the wild-type, 283, and 359 strains was 0.15, 0.78 and 0.64 g/L, respectively, indicating a fivefold and fourfold increase for the 283 and 359 strains over the wild-type strain, respectively (Table 3). In addition to higher maximum biomass, when compared with *C. vulgaris* ESP-31 wild type, mutants 283 and 359 have relatively higher maximum biomass productivity (125.76 and 94.79 mg/L/day), CO_2_ biofixation rate (236.42 and 178.20 mg/L/day) and specific growth rate (0.52 and 0.35 day^−1^).

**Table 3:** Maximum biomass, maximum biomass productivity, CO_2_ biofixation rate and specific growth rate of *Chlorella vulgaris* ESP-31 wild type and mutants under indoor and outdoor condition with simulated flue gas

Cultivation system	Strain	Maximum biomass (g/L)	Maximum biomass productivity (mg/L/day)	CO_2_ biofixation rate (mg/L/day)	specific growth rate (day^−1^)
Indoor with simulated flue gas	Wild type	0.15 ± 0.01	28.38 ± 1.83	53.35 ± 3.44	0.27 ± 0.02
	283	0.78 ± 0.02***	144.72 ± 0.17***	272.06 ± 0.31***	0.52 ± 0.07*
	359	0.64 ± 0.01***	103.21 ± 4.58**	194.03 ± 8.61**	0.35 ± 0.03
Outdoor with simulated flue gas	Wild type	0.53 ± 0.08	112.18 ± 10.66	210.89 ± 20.04	0.40 ± 0.11
	283	0.94 ± 0.10	243.15 ± 70.77	457.13 ± 133.05	0.55 ± 0.01
	359	0.89 ± 0.09	225.96 ± 27.48*	424.81 ± 51.66**	0.66 ± 0.23

Each datum indicates the mean ± SD from two experiments

* *P* < 0.05, **P < 0.01 and ***P < 0.001 were statistically significant as compared with *Chlorella vulgaris* ESP-31 wild type

When grown in simulated flue gas under outdoor culture conditions, the wild-type, 283, and 359 strains presented a maximum biomass of 0.53, 0.94 and 0.89 g/L, respectively, indicating a 1.8-fold and 1.7-fold increase for the 283 and 359 strains over the wild-type strain, respectively (Table 3). In addition to higher maximum biomass, compared with *C. vulgaris* ESP-31 wild type, mutants 283 and 359 have relatively higher maximum biomass productivity (243.15 and 225.96 mg/L/day), CO_2_ biofixation rate (457.13 and 424.81 mg/L/day) and specific growth rate (0.55 and 0.66 day^−1^).

These results clearly indicated that *C. vulgaris* ESP-31 mutants enhance both simulated flue gas and heat tolerances to the extent that they could successfully grow in closed outdoor PBR with simulated flue gas under severe high temperature and high illumination conditions.

## Discussion

Microalgae have been recognized as organisms for capturing CO_2_ in flue gases [25]. The cultivation of microalgae usually faces adverse effects of high temperature under outdoor culture conditions [26]. Therefore, *C. vulgaris* ESP-31 was subjected to NTG mutagenesis to obtain mutants tolerant to flue gas and high temperature. Two mutants exhibited the ability to tolerate simulated flue gases at 40 °C under indoor conditions, as evidenced by higher biomass, biomass productivity, and photosynthetic efficiency than that of the wild type.

The flue gases of thermal power plants in Taiwan have shown high CO_2_, NOx, and SOx emissions [27]. The current study showed that the high concentrations of CO_2_, NO and SO_2_ present in the flue gases had negative effects on the growth and CO_2_ sequestration processes of *C. vulgaris* ESP-31 wild type (net photosynthetic O_2_ evolution rate). The *C. vulgaris* ESP-31 mutants 283 and 359 could tolerate high concentrations of CO_2_, NO and SO_2_ present in the flue gases. These strains might be useful resources for CO_2_ sequestration and utilization of flue gases. Microalgae are considered to be organisms that can remove CO_2_, NOx, and SOx in flue gases [28]. Our present study showed that the mutants can grow well in simulated flue gas. This finding supported that these mutants could possibly use CO_2_, NO and SO_2_ for growth. Although we did not detect the concentrations of CO_2_, NO and SO_2_, we found that compared to the wild type, these mutants showed significant growth and photosynthetic efficiency under high CO_2_, NO, and SO_2_ concentrations. Our findings indicated that these mutants can utilize CO_2_, NO and SO_2_.

CO_2_ is a greenhouse gas that contributes to global warming [29]; it is mainly produced by burning fossil fuels and biomaterials and is emitted into the atmosphere as a global warming gas [30]. Algae production is well recognized as a solution for CO_2_ sequestration [31]. *Chlorella* spp., belonging to Chlorophyta, are freshwater and single-cell algae and represent one of the common algal species used for CO_2_ capture. The present study found that biomass production could reach 1.68, 1.91, and 1.99 g/L for the wild type and mutants 283 and 359, respectively, under 5% CO_2_ conditions (Fig. 1a), while comparatively, under 25% CO_2_ conditions, biomass production decreased, with values of 1.28, 1.61, and 1.54 g/L (Fig. 1b). A study examining the growth of the wastewater alga *C. vulgaris* ARC 1 in response to different CO_2_ concentrations ranging from 0.036 to 20% showed the highest biomass production of 0.21 g/L at 6% CO_2_ under a light intensity of 47 μmol m^−2^ s^−1^ [32]. The authors also found that CO_2_ concentration > 16% decreased the biomass production. This result was similar to our present results. A decrease in biomass was also found in *Chlorella* sp. KR-1 in response to high CO_2_ concentrations [33]. However, our data showed that even though the biomass decreased as the CO_2_ concentration increased in both mutants and the wild type, the mutants still retained a higher biomass than the wild type did. This result clearly demonstrated that these two mutants exhibited higher CO_2_ utilization ability.

*Chlorella vulgaris* ESP-31 wild type showed the highest biomass productivity of 0.029 g/L/day when 1200 mg/L NaHCO_3_ was used as the carbon source [34]. This value was lower than that from the present study using CO2 gas as the carbon source. This finding suggested that the growth of *C. vulgaris* ESP-31 wild type favoured CO2 as a carbon source compared to NaHCO3. In another previous study [21] using 2% CO2/air aeration, *C. vulgaris* ESP-31 wild type showed 0.26 g/L/day biomass productivity under indoor conditions. This value was also lower than that for *C. vulgaris* ESP-31 wild type cultured in the present study.

*Chlorella vulgaris* ESP-31 mutants 283 and 359 showed enhanced thermo-tolerance and 25% CO2 tolerance (Fig. 2). To further investigate the feasibility of using *C. vulgaris* ESP-31 mutants 283 and 359 for CO2 sequestration from industrial flue gas, *C. vulgaris* ESP-31 wild type and mutants 283 and 359 were grown at high temperature in medium aerated with simulated flue gas (25% CO2, 80–90 ppm SO2, 90–100 ppm NO) under both indoor (40 °C) and outdoor (~45 °C) conditions.

The maximum biomass, maximum biomass productivity, specific growth, CO_2_ biofixation rate, and specific growth rate of the mutants under the outdoor conditions were higher than those under the indoor conditions. It is obvious that the outdoor conditions were more suitable for algal cell growth. For example, the irradiance was higher in outdoor conditions and thus promoted algal cell growth.

The growth of microalgae under flue gas conditions not only varied between species but also was dependent on different flue gas compositions. In the present study, the maximum biomass of the *C. vulgaris* ESP-31 mutants 283 and 359 grown under outdoor conditions was 0.94 and 0.89 g/L, respectively, while the productivity was 243.15 and 225.96 mg/L/day. A *Chlorella* sp. cultured for 7 days with flue gas from burning coal containing 10% (v/v) CO_2_, 61 ppm NOx, and 0.3% (v/v) SOx showed maximum biomass and productivity values of 1.4 g/L and 191 mg/L/day, respectively [35]. Radmann et al. found that the cultivation of *Chlorella vulgaris* isolated from the coal-burning President Medici thermoelectric plant (UTPM, Candiota town, RS state, Brazil) using 12% (v/v) CO_2_, 60 ppm SO_2_ and 100 ppm NO in a serial tubular PBR showed maximum biomass and productivity values of 0.8 g/L and 80 mg/L/day, respectively [36].

In indoor cultivations, the wild type did not grow while the maximum biomass of the mutants 283 and 359 was 0.78 and 0.64 g/L, respectively, the maximum biomass productivity was 125.76 and 94.79 mg/L/day, the specific growth rates were 0.52 and 0.35 day^−1^, the CO_2_ biofixation rates were 236.42 and 178.20 mg/L/day, and the specific growth rates were 0.52 and 0.35 day^−1^. In contrast to the indoor condition, the wild type can grow under outdoor culture condition although the growth was smaller than mutants (Fig. 4a). Probably, high temperature of 40 °C maintained over the daytime in indoor condition while in outdoor condition, temperature remained low (< 40 °C) at the start of the daytime and then gradually increased to around 45 °C for approximately 2–3 h at noon, followed by a decrease in temperatures. Evidently, the exposure to high temperature in outdoor condition was shorter than the indoor condition. Under outdoor culture conditions the maximum biomass of the 283 and 359 mutants was 0.94 and 0.89 g/L, respectively, and the maximum biomass productivity was 243.15 and 225.96 mg/L/day. The CO_2_ biofixation rates were 457.13 and 424.81 mg/L/day, respectively, and the specific growth rates were 0.55 and 0.66 day^−1^.

The current study applied NTG mutagenesis (leading to transition mutations between GC and AT) together with high temperature and CO_2_ conditions to successfully obtain a mutant that can grow in simulated flue gas. In fact, microalgal species tolerant to flue gas can be obtained through the adaptive evolution method. The use of real flue gas injection into the microalgal community for a long time can also successfully select the species that can grow under flue gas conditions [37]. A recent study by Cheng et al. revealed that a new strain, *Chlorella* sp. Cv, can be obtained through adaptive evolution (46 cycles) against simulated flue gas (10% CO_2_, 200 ppm NOx and 100 ppm SOx) with a maximum CO_2_ fixation rate of 1200 mg/L/day [38]. This value was higher than that of our outdoor results, in which the maximum CO_2_ biofixation rates of *C. vulgaris* ESP-31 mutants 283 and 359 were 457.13 and 424.81 mg/L/day, respectively. It is evident that the CO_2_ utilization ability of microalgae was higher for the species selected by adaptive selection.

Microalgal growth is known to fix CO2 from flue gas and can also use NOx and SOx as nutrients for their growth [9]. According to our results that were obtained by using NO and SO_2_, we did not know whether the mutants obtained in the present study can use or tolerate NOx and SOx. A study similar to our present work was also using simulated flue gas containing CO_2_, SO_2_, NO and ash for adaptive evolution selection of the algal species that can grow in simulated flue gas [39]. However, further study is needed to identify the tolerance of the *C. vulgaris* ESP-31 mutant to real flue gas containing CO_2_, NOx, SOx and ash.

SO_2_ was much more dangerous than NO. The presence of SO_2_ at high concentrations can inhibit the growth of microalgae [40] due to a decrease in pH [41]. Our findings showed that the injection of simulated flue gas containing 80–100 ppm SO_2_ promoted the growth of the *C. vulgaris* ESP-31 mutants. Furthermore, microalgal growth was not affected by 50 ppm SO2 but was significantly affected by 400 ppm SO2 due to a decrease in pH [41]. Evidently, the 80–90 ppm SO2 used in the present study was not high enough to inhibit algal growth. Several studies have also shown *Chlorella* spp. can tolerate high SO_2_ gas levels. Duarte et al. found that *Chlorella fusca* LEB 111 showed no inhibition of cell growth with 400 ppm SO_2_ gas present in the flue gas from a coal power plant [39]. Using the injection of flue gas every 2 h of the light period (between 7 and 19 h) for 10 min through a porous curtain sparger fixed on the PBR base, *Chlorella fusca* LEB 111 can even grow in the flue gas containing SOx (5000 ppm) as well as CO_2_ (10–12%, v/v), NOx (400 ppm), and ash (650 ppm) [14].

The impact of SO_2_ on microalgal growth may be due to a drop in the pH caused by SOx injection in the waters [42]. Although our present study did not determine the medium pH, it would be expected that the medium pH under simulated flue gas conditions did not drop during the culture of the mutant, possibly due to the utilization of CO_2_ by the mutants with normal photosynthetic ability. However, for the control cell, the photosynthetic ability was significantly decreased, and as a result, the pH was decreased by SO_2_ injection; hence, cell growth was inhibited. Further study is needed to prove this hypothesis in the future.

SO_2_ may exert different effects in different temperature condition. It is because the solubility of SO_2_ was lower under 40 °C than that under 28 °C. Therefore, under indoor condition, the concentration of SO_2_ in night time was higher than the daytime period As a result, SO_2_ in night time can be much more utilized than that in the daytime. This can be also found in the outdoor conditions.

In addition to the increase in growth rate, a significant increase in the production of carbohydrates and lipids in mutants 283 and 359 grown under flue gas and high-temperature conditions was observed in both indoor and outdoor cultivations. Carbohydrates can be utilized for fermentation into ethanol as fuel energy. However, the lipid content of the wild type and 283 and 359 mutants was lower than that in a previous study using *C. vulgaris* ESP-31 as a material [21]. Besides, the present data from lipid content and Nile red stain showed that the wild type cultured did not accumulate lipid under indoor condition, but can accumulate under outdoor condition. It was possible because of the death of algal cells under indoor condition but the maintenance of growth under outdoor condition. These results indicated that flue gases and/or high temperature exhibited a negative effect on lipid accumulation in the lipid-rich green microalga, *C. vulgaris* ESP-31.

Obviously, the photosynthetic activity (photosynthetic O2 evolution rate and PSII activity) was increased after mutation for enhanced CO2 utilization in *C*. *vulgaris* ESP-31 in CO2-enriched flue gas. Photosystem II biogenesis is important in cyanobacteria [43]. The results suggest that the efficiency of the photosystem is enhanced in the mutants and therefore improves the light energy input used for CO2 fixation. Thus, the biomass is increased under increased CO2 supplementation.

High temperature (HT) stress inhibits photosynthetic machinery, and the primary sites of targets of high temperature stress are photosystem II (PSII) by increasing the fluidity of thylakoid membranes at high temperature, which causes dislodging of PSII light-harvesting complexes on the thylakoid membrane and affects electron dynamics depending on PSII integrity [44]. High temperature also impacts ribulose-1,5-bisphosphate carboxylase/oxygenase (Rubisco) kinetics and induces photorespiration [44]. A higher PSII activity and photosynthetic O2 evolution rate in the mutants demonstrated that photosynthesis in *C. vulgaris* ESP-31 wild type decreased upon exposure to high temperature, while the mutants could overcome the inhibitory effect of high temperature. It is obvious that enhanced CO2 sequestration was achieved in these mutant strains. In addition, although we did not exactly study the tolerance mechanism in these mutants, it has been suggested that major tolerance mechanisms, including ion transporters, increased production of late embryogenesis abundant (LEA) proteins, accumulation of heat shock proteins (HSPs) and osmoprotectants, and involvement of the antioxidant defence system are essential to cope with high temperature stress [44].

## Conclusions

The present study reported the isolation and characterization of two thermo-tolerant and high CO_2_-tolerant mutants of *C. vulgaris* ESP-31, namely, mutants 283 and 359. These two mutants gained the ability to directly and efficiently grow in simulated flue gas (25% CO_2_, 80–90 ppm SO_2_, 90–100 ppm NO) by using outdoor PBR. The results also revealed that the photosynthetic activity (photosynthetic O_2_ evolution rate and PSII activity) of mutants 283 and 359 increased after mutation for enhanced CO_2_ utilization in *C*. *vulgaris* ESP-31. These strains represent promising microalgae for sequestration and utilization of CO_2_ from flue gases using outdoor PBR.

## Methods

### Microalgae cultivation

*Chlorella vulgaris* ESP-31 was obtained from Prof. Jo-Shu Chang, Department of Chemical Engineering, National Cheng-Kung University, Tainan, Taiwan [34]. *Chlorella vulgaris* ESP-31 cells were cultured in 50 mL BG-11 medium, pH 7.4 [45]. The cultures were normally incubated at 28 °C under continuous light conditions (approximately 50 μmol m^−2^ s^−1^, illuminated by a TL5 lamp (Philips, Singapore). The light intensity was measured by a Li-250A Light Meter with a Li-190 quantum sensor (Li-COR Inc., Lincoln, Nebraska, USA).

### NTG mutagenesis

To isolate heat-tolerant mutants of *C. vulgaris* ESP-31, the wild-type strain was mutagenized with *N*-methyl-*N*′-nitro-*N*-nitrosoguanidine (NTG) [46]. First, 30 mL log-phase cultures of strain *C. vulgaris* ESP-31 were harvested and resuspended in 10 mL 10-mM citrate buffer, pH 6.0. These cells were then treated with NTG (500 μg/mL) for 30 min at room temperature in the light. The treated cells were washed three times with 10 mL BG-11 medium and then harvested by centrifugation. The survivors were plated onto BG-11 plates at 40 °C and grown under continuous light. The colonies that grew better on plates at 40 °C were picked and then restreaked onto fresh BG-11 plates.

### Screening of *Chlorella vulgaris* ESP-31 mutants with the ability to tolerate heat and high levels of CO_2_

**Fig. 5:**
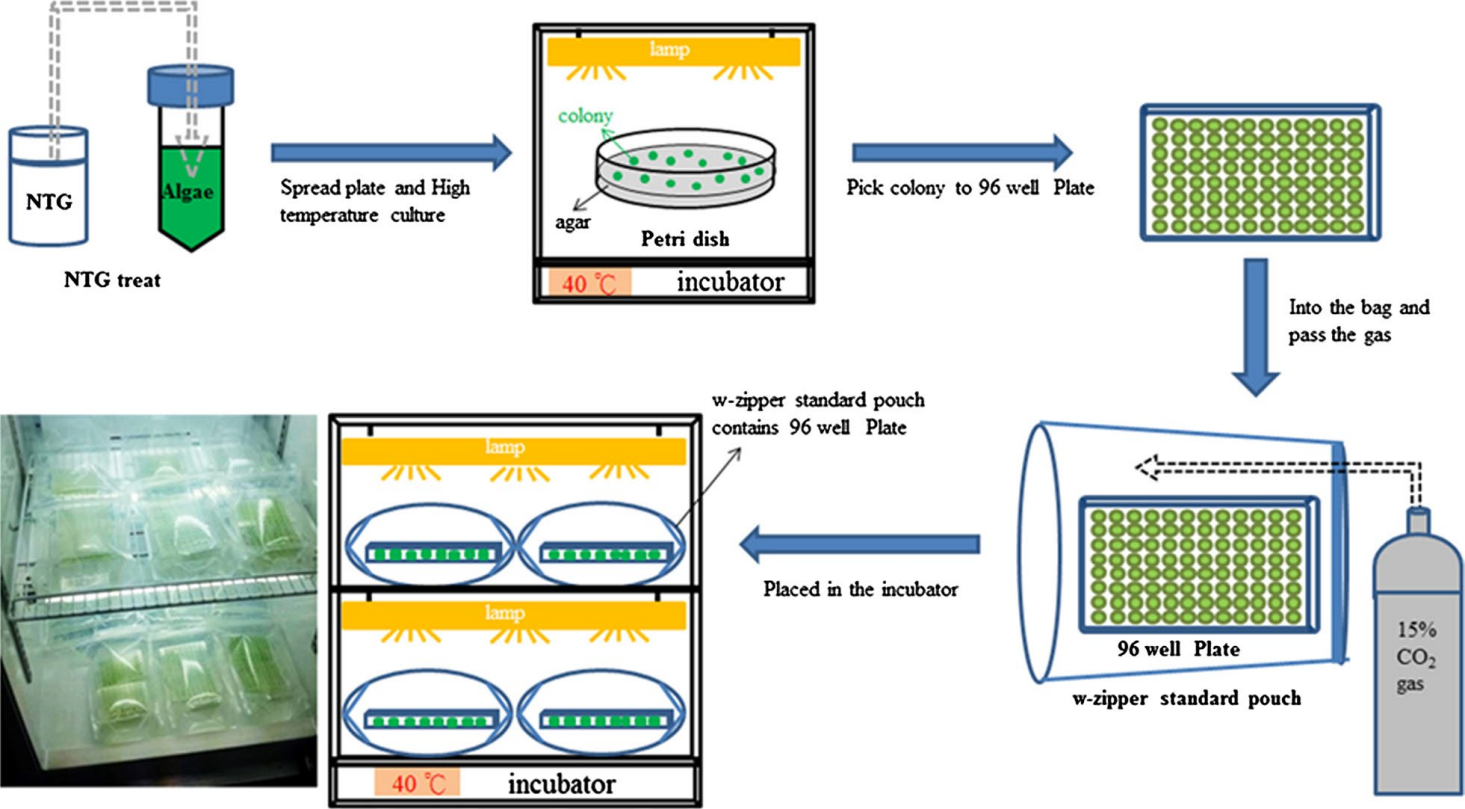
Schematic representation of NTG mutagenesis and screening of *Chlorella vulgaris* ESP-31 mutants for high temperature tolerance and high CO_2_ concentration tolerance

To screen the thermo-tolerant and high CO_2_ concentration-tolerant mutants of *C. vulgaris* ESP-31, a 48-well microtitre plate (without lid) containing multiple mutant strains was placed inside a w-zipper standard pouch (C-43, Mitsubishi Gas Chemical Company, Inc., Japan), and the bag was aerated with 15% CO_2_/air. The sealed bags were incubated at 40 °C and illuminated with 50 μmol m^−2^ s^−1^. Growth was monitored by measuring the OD_680_ every 24 h using a Synergy HT Multi-Detection Microplate Reader (BioTek Instruments, Inc., USA). After the reading, the culture plates were placed back into the bags and re-charged with 15% CO_2_/air (the process is shown in Fig. 5).

### Growth of cells under various CO_2_ concentrations and high temperature stress conditions

*Chlorella vulgaris* ESP-31 wild type and mutant cells were collected and diluted to an OD_680_ of 0.2 with 50 mL freshly prepared BG-11 medium (containing 1.5 g/L NaNO_3_). Cells were cultivated at 28 °C with a speed of rocking at 150 rpm and a light/dark cycle of 12 h/12 h at a light intensity of 150 μmol m^−2^ s^−1^. For growth experiments in different CO_2_ concentrations, cells were cultivated by aeration with 5% and 25% CO_2_ (0.1 vvm), separately. For growth experiments under high temperature stress, cells were cultivated in an incubator at 12 h light (40 °C)/12 h dark (28 °C) by aeration with 25% CO_2_ (0.1 vvm). Growth was determined by measuring the OD at 680 nm using a spectrophotometer (CT-5600, ChromTech, USA) every 24 h.

### Growth of cells under indoor simulated flue gas conditions

*Chlorella vulgaris* ESP-31 wild type and mutant cell pre-cultures were normally incubated at 28 °C under continuous light conditions (approximately 50 μmol m^−2^ s^−1^, illuminated by a TL5 lamp). Then, the cultures were collected and diluted to an OD_680_ of 0.4 with 800 mL freshly prepared ¼ N BG-11 medium (containing 0.375 g/L NaNO_3_) in a 1 L PBR (length × i.d. = 22 × 8 cm). In the intermittent flue gas aeration, culture aeration was controlled by a gas switch, and a gas-switching cycle was performed with simulated flue gas containing 25% CO_2_/air, 80–90 ppm SO_2_, 90–100 ppm NO at a rate of 0.1 vvm. The temperature was controlled at 40 °C with a rocking speed of 150 rpm under a light cycle consisting of 12 h of light with 150 μmol m^−2^ s^−1^ on single sides of the bottle and then aeration with air (0.1 vvm) at 28 °C with a rocking speed of 150 rpm for 12 h of dark for 9 days. Then, the cells were cultivated by aeration with air (0.1 vvm) at 28 °C with a rocking speed of 150 rpm and a light/dark cycle of 12 h/12 h at a light intensity of 150 μmol m^−2^ s^−1^ until 28 days. Growth was monitored by measuring the OD_680_ every 24 h.

### Growth of cells in a closed PBR with simulated flue gas under outdoor culture conditions

*Chlorella vulgaris* ESP-31 wild type and mutant cells were collected and diluted to an OD_680_ of 0.4 with 1.8 L freshly prepared ¼ N BG-11 medium (containing 0.375 g/L NaNO_3_) in a 2 L PBR (length × i.d. = 25 × 12 cm). In the intermittent flue gas aeration, culture aeration was controlled by a gas switch, and a gas-switching cycle was performed with simulated flue gas containing 25% CO_2_/air, 80–90 ppm SO_2_, 90–100 ppm NO (0.1 vvm) at daytime and then aeration with air (0.1 vvm) at night for 6 days. Then, cells were cultivated by aeration with air (0.1 vvm) until 30 days. The outdoor PBR cultivations lasted from September 6–October 6, 2017. Growth was monitored by measuring the OD_680_ every 24 h.

### Determination of chlorophyll content

To determine the chlorophyll content, 1 mL algal cell culture was centrifuged at 12,000×*g* at 4 °C for 5 min, and the pellet was ground in liquid nitrogen and extracted with 1 mL chilled 95% ethanol at 4 °C for 10 min. After centrifugation at 12,000×*g* at 4 °C for 5 min, the supernatant was collected, and the volume was adjusted to 1 mL with 95% ethanol. The absorbance of the supernatant was detected at 645 nm and 663 nm for the estimation of the chlorophyll content. The contents of chlorophyll, chlorophyll *a*, and chlorophyll *b* were determined by the following equations [47]:

1 $$ {\text{Chl}}a\left( {\upmu {\text{g}}/{\text{mL}}} \right) \, = { 12}. 7 { }\left( {{\text{A}}_{ 6 6 3} } \right) \, {-}{ 2}. 5 9 { }\left( {{\text{A}}_{ 6 4 5} } \right), $$

2 $$ {\text{Chl}}b\left( {\upmu {\text{g}}/{\text{mL}}} \right) \, = { 22}. 9 { }\left( {{\text{A}}_{ 6 4 5} } \right) \, {-}{ 4}. 6 7 { }\left( {{\text{A}}_{ 6 6 3} } \right), $$

3 $$ {\text{Chl }}\left( {\upmu {\text{g}}/{\text{mL}}} \right) \, = {\text{ Chl}}a + {\text{ Chl}}b. $$

### Measurements of photosynthetic oxygen evolution rate

Photosynthesis was determined by detecting the amount of O_2_ evolution and consumption by algal cells using a Clark-type oxygen electrode fitted with a DW3 chamber (Hansatech, King’s Lynn, Norfolk, England) thermostat at 28 °C. The cell culture was centrifuged at 12,000×*g* for 5 min at room temperature. The cells were resuspended in 3 mL 50-mM HEPES buffer (pH 7.4) containing 5 mM NaHCO_3_. The O_2_ evolution of net photosynthesis was determined in 300 μmol m^−2^ s^−1^ for 8 min. The net photosynthesis rate was expressed as the change in O_2_ concentration per hour, as previously described [48]. Two replicates per treatment were measured.

### Chlorophyll *a* fluorescence determination of maximum PSII activity using pulse amplitude modulation (PAM) fluorometry

Chlorophyll *a* fluorescence parameters were employed to determine the activity of photosystem II (PSII) using an AP-C 100 system (AquaPen, Brno, Czech Republic). An aliquot of the algal culture was diluted with BG-11 medium to an OD_680_ = 0.2. A 2-mL aliquot of diluted algal cells was then transferred to an AquaPen cuvette and subjected to a pulse of saturating light of 4000 μmol m^−2^ s^−1^ PAR to obtain the light-adapted minimal fluorescence (*F*_t_) and the light-adapted maximal fluorescence (*F*_m_’). To determine the maximum PSII activity, *F*_v_/*F*_m_ (=*F*_m_ − *F*_o_/*F*_m_), 2 mL diluted algal cell suspension in an AquaPen cuvette was incubated in the dark for 30 min and then flushed with saturated light (4000 μmol m^−2^ s^−1^) to obtain the dark-adapted minimal fluorescence (*F*_o_) and dark-adapted maximal fluorescence (*F*_m_). The maximum PSII activity, *F*_v_/*F*_m_ = *F*_m_ − *F*_o_/*F*_m_, was then calculated.

### Nile red staining and microscopy

To detect lipid droplets, Nile red was used to stain the wild type and mutants 283 and 359 of *C. vulgaris* ESP-31. An aqueous solution of Nile red of 0.1 μg/mL was incubated for approximately 10 min in the dark at room temperature [49, 50]. After staining, Nile red fluorescence and phase contrast micrographs were taken on a LEICA microscope (LEICA DM2500) with 100× objective using specific emission filter sets (bypass excitation (475 nm) and longpass emission (510 nm) filters).

### Determination of carbohydrate content of *Chlorella vulgaris* ESP-31

The carbohydrate content in the *C. vulgaris* ESP-31 wild type and mutants was determined using the modified quantitative saccharification (QS) method reported by the Nation Renewable Energy Laboratory (NREL), USA [51]. A small amount of dry cell powder was added to 1 mL 72% (v/v) sulfuric acid and incubated for 60 min at 30 °C for primary hydrolysis. The hydrolysate was then diluted to 4% (v/v) with sulfuric acid and incubated at 121 °C (sterilization) for 20 min as the secondary hydrolysis. The supernatant was neutralized and analysed by high-performance liquid chromatography for sugar assays [52].

### Determination of lipid content

The biomass of *Chlorella vulgaris* ESP-31 wild type and mutants was harvested by centrifugation (6000 rpm for 10 min) and then washed twice with deionized water and lyophilized. The lipid content of cells was determined as fatty acid methyl esters (FAMEs) through the direct transesterification method. The samples were analysed by gas chromatography (GC-2014, Shimadzu, Kyoto, Japan) equipped with a flame ionization detector (FID) [34, 53, 54].

### Determination of growth biomass productivity and specific growth rate

The biomass productivity (*P*, mg/L/day) was calculated from the change in biomass concentration (*X*, mg/L) within a culture time (*t*, d) according to the equation *P* = (*X*_1_ − *X*_0_)/(*t*_1_ − *t*_0_). The specific growth rate *μ* (day^−1^) was calculated from the equation *μ *= ln (*X*_1_ − *X*_0_)/(*t*_1_ − *t*_0_), where *X*_1_ and *X*_0_ represented the biomass concentration (mg L^−1^) on days *t*_*1*_ and *t*_0_, respectively [28].

### Determination of CO_2_ biofixation rate

The fixation rate of CO_2_ (mg/L/day) was calculated from the equation CO_2_ fixation rate = 1.88 × *P*, where *P* is the biomass productivity (mg/L/day) [57].

### Statistical analysis

Experimental data were calculated and expressed as the mean ± SE. Duncan’s new multiple range was used for the test of the data among the wild type and two mutants. A difference was considered to be statistically significant when *P* < 0.05.

## Data Availability

The datasets supporting the conclusions of this article are included within the article.

## Abbreviations

PBR: photobioreactor
HT: high temperature
FAMEs: fatty acid methyl esters
FID: flame ionization detector
*F*_m_: maximal fluorescence
*F*_o_: minimal fluorescence
HSPs: heat shock proteins
LEA: late embryogenesis abundant
NTG: *N*-methyl-*N*′-nitro-*N*-nitrosoguanidine
PAM: pulse amplitude modulation
PSII: photosystem II
Rubisco: ribulose-1,5-bisphosphate carboxylase/oxygenase
WT: wild type
